# Hypoxic mesenchymal stem cell-derived extracellular vesicles ameliorate renal fibrosis after ischemia–reperfusion injure by restoring CPT1A mediated fatty acid oxidation

**DOI:** 10.1186/s13287-022-02861-9

**Published:** 2022-05-07

**Authors:** Zhumei Gao, Chuyue Zhang, Fei Peng, Qianqian Chen, Yinghua Zhao, Liangmei Chen, Xu Wang, Xiangmei Chen

**Affiliations:** 1 Department of Nephrology, First Medical Center of Chinese PLA General Hospital, Nephrology Institute of the Chinese People’s Liberation Army, State Key Laboratory of Kidney Diseases, National Clinical Research Center for Kidney Diseases, Beijing Key Laboratory of Kidney Disease Research, Beijing, China; 2 Department of Nephropathy, The Second Hospital of Jilin University, Changchun, China; 3 Kidney Research Institute, National Clinical Research Center for Geriatrics and Division of Nephrology, West China Hospital of Sichuan University, Chengdou, China; 4 School of Clinical Medicine, Tsinghua University, Beijing, China; 5 Department of Nephrology, The First Affiliated Hospital of Jinan University, Guangzhou, China

**Keywords:** Mesenchymal stem cell, Hypoxic, Extracellular vesicles, Mitochondrial, Fatty acid oxidation, Renal fibrosis

## Abstract

**Background:**

Renal fibrosis is a common pathological process of chronic kidney diseases induced by multiple factors. Hypoxic pretreatment of mesenchymal stem cells can enhance the efficacy of secreted extracellular vesicles (MSC-EVs) on various diseases, but it is not clear whether they can better improve renal fibrosis. The latest research showed that recovery of fatty acid oxidation (FAO) can reduce renal fibrosis. In this study, we aimed to examine whether hypoxic pretreatment with MSC extracellular vesicles (Hypo-EVs) can improve FAO to restore renal fibrosis and to investigate the underlying mechanism.

**Methods:**

Hypo-EVs were isolated from hypoxia-pretreated human placenta-derived MSC (hP-MSC), and Norm-EVs were isolated from hP-MSC cultured under normal conditions. We used ischemia–reperfusion (I/R)-induced renal fibrosis model in vivo. The mice were injected with PBS, Hypo-EVs, or Norm-EVs immediately after the surgery and day 1 postsurgery. Renal function, kidney pathology, and renal fibrosis were assessed for kidney damage evaluation. For mechanistic exploration, fatty acid oxidation (FAO), mitochondrial morphological alterations, ATP production and mitochondrial mass proteins were detected in vivo. Mitochondrial membrane potential and reactive oxygen species (ROS) production were investigated in vitro.

**Results:**

We found that Hypo-EVs confer a superior therapeutic effect on recovery of renal structure damage, restoration of renal function and reduction in renal fibrosis. Meanwhile, Hypo-EVs enhanced mitochondrial FAO in kidney by restoring the expression of a FAO key rate-limiting enzyme carnitine palmitoyl-transferase 1A (CPT1A). Mechanistically, the improvement of mitochondrial homeostasis, characterized by repaired mitochondrial structure, restoration of mitochondrial mass and ATP production, inhibition of oxidative stress, and increased mitochondrial membrane potential, partially explains the effect of Hypo-EVs on improving mitochondrial FAO and thus attenuating I/R damage.

**Conclusions:**

Hypo-EVs suppress the renal fibrosis by restoring CPT1A-mediated mitochondrial FAO, which effects may be achieved through regulation of mitochondrial homeostasis. Our findings provide further mechanism support for development cell-free therapy of renal fibrosis.

**Supplementary Information:**

The online version contains supplementary material available at 10.1186/s13287-022-02861-9.

## Background

Chronic kidney disease (CKD) is a severe disease threatening human health and is also an important global public issue. The global incidence, prevalence, and death of CKD are significant, rising, and the prevalence is approximately 10% among adults of worldwide [1, 2]. Renal ischemia reperfusion (I/R) injury is associated with renal fibrosis and progressive CKD [3, 4]. Renal fibrosis is considered a common pathological process that leads to CKD and end-stage renal disease. However, there are currently no effective therapies that successfully prevent or reverse fibrosis [5].

Mesenchymal stem cell-derived extracellular vesicles (MSC-EVs) have been reported to play a regenerative and antifibrotic role in several preclinical models of CKD [6–9]. However, the low yield of MSC-EVs is a limiting factor for large-scale production of cell-free therapies [10]. Hypoxia preconditioning stimulates the paracrine activities of MSCs and increases the function and production of EVs and is considered an engineering approach to improve the therapeutic potential of EVs [11, 12]. A recent study indicated that hypoxia-preconditioned MSCs are more significant than untreated MSCs in preventing renal fibrosis and inflammation [13]. However, the role of hypoxia-preconditioned MSC-derived EVs in renal fibrosis has not yet been established.

The pathological mechanism of renal fibrosis has been reviewed in many recent studies [14–16], particularly in the field of metabolic regulation [17]. It is well known that proximal tubular cells (PTCs) are the most energy-demanding cells in the kidney and play a central role in tubulointerstitial fibrosis progression [18, 19]. Fatty acid oxidation (FAO) is the primary energy source of PTCs [19, 20]. The results of genome-wide transcriptome studies showed that the expression of FAO-related key metabolic enzymes and transcription regulators were markedly decreased in humans and mouse models with tubulointerstitial fibrosis [20]. In addition, restoring fatty acid metabolism can protect mice from tubulointerstitial fibrosis [17, 20]. A recent study confirmed that overexpression the key rate-limiting enzyme carnitine palmitoyl-transferase 1A (CPT1A) can enhance FAO in renal tubular epithelial cells, reduce the expression of inflammatory mediators, and lead to a significant alleviation of fibrosis in three experimental models [21]. Therefore, FAO-targeted therapies may be a potentially effective therapeutic strategy for fibrosis reduction.

Mitochondria play an indispensable role in regulating FAO. A previous study indicated that astragaloside IV can enhance FAO by regulating mitochondrial activity during ageing [22]. The destruction of mitochondrial homeostasis plays an important role in the early injury of kidney disease, abnormal repair after injury and the pathogenesis of CKD [23, 24]. Under physiological and pathological conditions, various mechanisms coordinate to maintain mitochondrial homeostasis, including antioxidant defence, mitochondrial DNA repair, mitochondrial fusion and fission, mitochondrial autophagy and mitochondrial biogenesis [25]. Several studies have shown that MSC-EVs can attenuate mitochondrial damage by stabilising mitochondrial DNA [26], inhibiting mitochondrial fission, and stimulating mitochondrial antioxidant defence and adenosine triphosphate (ATP) production in acute kidney injury (AKI) models [27, 28]. Previous studies indicated that Hypo-EVs treatment had better effects on promoting angiogenesis, proliferation and migration, and inhibiting inflammation and apoptosis than Norm-EVs treatment [29–31]. Nevertheless, researchers have not conclusively determined whether the therapeutic outcomes of Hypo-EVs in fibrosis depend on mitochondrial homeostasis.

The aims of this study were (1) to explore whether hypoxic pre-treatment of MSCs derived extracellular vesicles(Hypo-EVs) has a better anti-fibrosis effect; (2) to determine whether Hypo-EVs alleviate ischemia reperfusion-induced fibrosis by enhancing CPT1A-mediated FAO; (3) to examine whether the protective effect of Hypo-EVs on FAO is related to the regulation of mitochondrial homeostasis.

## Methods

### Cell culture and hypoxia preconditioning

Human placenta-derived MSC (hP-MSC) were provided by the Institute of Foundation, Chinese Academy of Medical Sciences and cultured in Dulbecco’s Modified Eagle’s Medium/Nutrient Mixture F-12 (DMEM/F12, Gibco) medium with 10% foetal bovine serum (FBS, Corning) supplemented with 100 U/mL penicillin − streptomycin (Gibco) and maintained at 37 °C in a humidified 5% CO_2_ atmosphere. After the hP-MSC reached 60%–70% confluency, the culture media were replaced with exosome-depleted FBS (SBI, 50A-1) and were cultured at 37 °C, 5% CO_2_, 21% O_2_ or at 1% O_2_, 94% N_2_, and 5% CO_2_ for an additional 48 h. The medium was then collected for EVs isolation, and the EVs collected from the hypoxic pretreatment of MSC (1% O_2_) were called Hypo-EVs, and the normal MSC (21% O_2_) were called Norm-EVs. Only hP-MSC from passages 6–10 were used for further experiments.

### Cell culture and treatment

Human renal proximal tubular epithelial (HK-2, ATCC, USA) cells were cultured in DMEM/F12 with 10% FBS and supplemented with 100 U/mL penicillin − streptomycin. The cells were maintained at 37 °C in a humidified atmosphere containing 5% CO_2_. We used two approaches to mimic CKD in vitro. The cell hypoxia/reoxygenation (H/R) model was performed as previously reported [32]. Briefly, HK-2 cells were exposed to a hypoxic condition (37℃, 1% O_2_, 94% N_2_, and 5% CO2) for 12 h in glucose- and serum-free medium to induce hypoxic injury. Subsequently, the medium was replaced, and the cells were placed under the normal condition (21% O2) for reoxygenation 24 h. The control group was cultured under the normal condition (21% O2) according to the experimental design. Another method was to incubate HK-2 cells with transforming growth factor beta-1 (TGF-β1) at a final concentration of 10 ng/mL for 48 h as previously reported [13]. During reoxygenation or TGF-β1-stimulated time Norm-EVs or Hypo-EVs (100 μg/mL of nutrient medium) were added to the cells.

### MSC-EVs isolation and identification

MSC-derived EVs were isolated using a differential ultracentrifugation method. Briefly, the culture medium was centrifuged at 4 °C as follows: 500 × g for 10 min, at 2,000 × g for 20 min, and at 10,000 × g for 30 min. The medium was filtered through a 0.22-μm filter before ultracentrifugation at 100,000 × g for 2 h. Finally, the EV pellets were resuspended in PBS and ultracentrifuged for an additional 2 h to discard the contaminating proteins, and then the purified EVs were harvested in PBS and stored at − 80 °C for further use. The protein concentrations of the Norm-EVs and Hypo-EVs were examined using a bicinchoninic acid protein assay (BCA, Thermo Fisher Scientific, Waltham, MA, USA). The typical sphere-shaped bilayer membrane structure of the EVs was observed using transmission electron microscopy (TEM). Western blotting was used to examine the quality of the EVs, and the size distribution of the EVs was examined by dynamic light scattering (DLS).

### Animal experiments

**Fig. 1:**
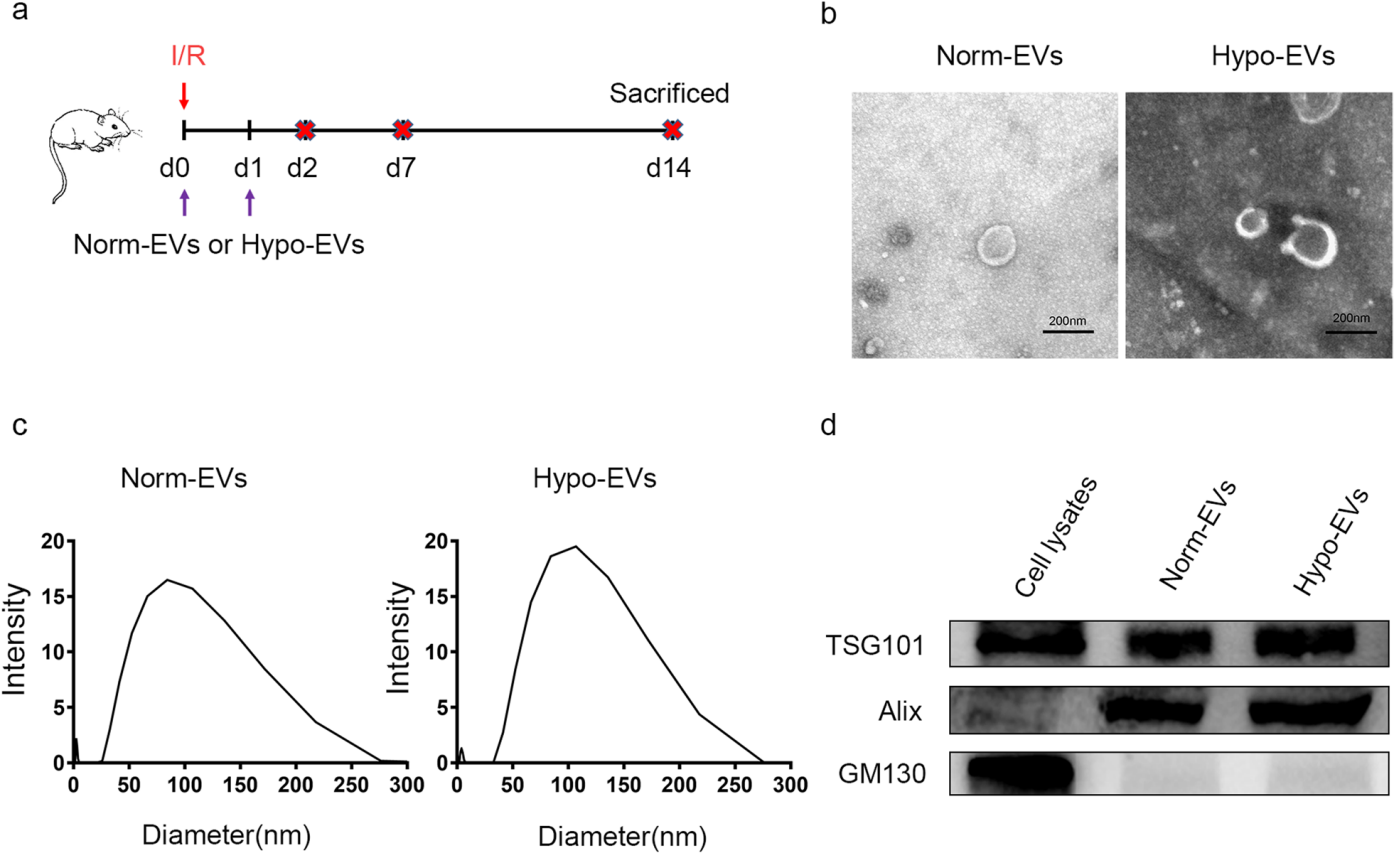
Characterization of EVs. **a** Schematic diagram of the animal experiment. Briefly, mice were treated with Norm-EVs (100 μg), Hypo-EVs (100 μg) or PBS immediately after surgery and on D 1 postsurgery and were sacrificed at D 2, D 7, and D 14 after surgery. **b**Transmission electron microscopy image of both analysed EVs. Scale bar represents 200 nm. **c** Dynamic light scattering measuring the size distribution of both analysed EVs. **d** Western blot analysed the EVs-specific positive biomarkers (Alix and TSG101) and the negative marker GM130 in the EVs and their parent cell (MSC)

Male C57BL/6 SPF mice (7–9 weeks old) were supplied by the Sibeifu Experimental Animal Science and Technology (Beijing, China) and were fed adaptively for 4 d in the animal centre at the Chinese PLA General Hospital before the experiment. The mice were randomly divided into four groups: the control, phosphate-buffered saline (PBS), Norm-EVs, and Hypo-EVs groups. Mice of PBS, Norm-EVs, and Hypo-EVs groups were subjected to renal ischemia reperfusion as described previously [33, 34]. Briefly, mice were anesthetized with pentobarbital (50 mg/kg) through intraperitoneal injection. The dorsal hair above the mouse kidney was cleaned and then the bilateral renal pedicle vessels were exposed. In order to induce renal ischemia, the left renal artery was clamped with a nontraumatic vascular clamp for 30 min and the right kidney was removed simultaneously. Afterwards, reperfusion was initiated by releasing the clamp. The kidneys were observed for 5 min to ensure reperfusion after clamp were removed. Operating table and room temperature were maintained at 37 °C during surgery. In treatment models, 100 μg of Norm-EVs or Hypo-EVs in 0.15 mL of PBS solvent was administered by tail vein injection immediately after the surgery and day (D) 1 postsurgery in the Norm-EVs and Hypo-EVs groups, respectively. The same volume of PBS was administered by tail vein injection in the PBS group. The mice were killed at D 2, D 7, and D 14 after surgery (Fig. 1a).

### MSC-EVs internalization

To verify whether EVs could be internalized by injured kidney. Dil (Yeasen) fluorescent staining was performed to label the EVs. The Norm-EVs or Hypo-EVs (200 μg) that were dissolved in 100 μL of PBS were incubated with 4 μM of Dil at 37 °C for 1 h. The unbound dyes were then removed using an exosome spin column (Invitrogen). The Di/Norm-EVs and Dil/Hypo-EVs were suspended in PBS for subsequent use. Dil/Norm-EVs or Dil/Hypo-EVs(100 μg) were intravenously injected into an ischaemia–reperfusion injury (I/R) model mice via the tail vein immediately after the surgery and on D 1 postsurgery as mentioned above. On days 1, 3, 5, and 7, the mice were imaged immediately using Living Image Software 4.4 (PerkinElmer). Moreover, the kidneys were collected and observed on an optical imaging system on D 7. All bioluminescence imaging (BLI) signals were measured by the average radiance from the regions of interest (ROIs).

### Transmission electron microscope(TEM)

A total of 1 mm3 of kidney tissue was fixed in 2.5% glutaraldehyde (pH 7.4) at 4℃ for 24 h. Then, the tissue was fixed with 1% osmium acid for 2–3 h, dehydrate it with acetone and ethanol, and embedded it with epoxy resin. After that, an ultramicrotome was used to prepare ultrathin sections with a thickness of 60 nm, which were then stained with 3% uranyl acetate and lead citrate. Finally, the section were observed under a TEM (Japan).

### Renal function analysis

A blood sample was collected from the tail vein on D 3, and the levels of blood urea nitrogen (BUN) in the mice were tested using an appropriate kit (Bioassay).

### Renal histopathology assessment

Renal tissues were fixed in 10% formalin for 48 h, embedded in paraffin, sectioned at 3 μm thickness, stained with Masson staining reagents by a standard protocol, observed using a microscope (Olympus, Tokyo, Japan). The percentage of collagen deposition area was calculated using ImageJ software. There were at least 10 random non-overlapping fields per animal for scoring.

### Immunohistochemistry

The paraffin-embedded kidney sections were pretreated using heat-mediated antigen retrieval with sodium citrate buffer (pH 6, epitope retrieval solution 1) for 20 min, followed by incubation with primary antibodies overnight at 4 °C. The sections were then incubated with a biotinylated secondary antibody, and 3,3’-diaminobenzidine (DAB) was used as the chromogen. The sections were then counterstained with haematoxylin and observed with optical microscope. The primary antibodies used were as follows: CPT1A (ab128568, Abcam), Vimentin (ab92547, Abcam).

### Immunofluorescence

Cells or tissue were permeabilised with 0.2% Triton X-100 for 5 min, blocked with sheep serum for 30 min, and incubated with α-smooth muscle actin (α-SMA, 1:200, 55135, Proteintech) or collagen I (1:100, ab270993, Abcam) antibody for 16–18 h at 4 °C, followed by incubation with Cy3‐conjugated secondary antibody (red) at room temperature for 1 h. Nuclei was counterstained with DAPI. Fluorescent staining of the tissue sections were imaged by confocal fluorescence microscopy.

### Western blotting analysis

The kidneys, cells, or EVs pellets were lysed in Radio-Immune Precipitation Assay (RIPA) lysis buffer containing phenylmethylsulphonyl fluoride (PMSF), and the supernatant suspension obtained after centrifugation was measured using a BCA protein assay kit (Thermo Fisher Scientific). Protein samples of the same quality (20 μg/lane) were added to a 10% or 12% sodium dodecyl sulphate–polyacrylamide gel (SDS-PAGE) and then transferred from the SDS-PAGE gels to the membranes. The membrane was blocked for 2 h at room temperature with a 1X casein solution, and then incubated with primary antibodies at 4 °C against the following proteins: α-SMA (ab7817, Abcam), vimentin (ab92547, Abcam), glyceraldehyde 3-phosphate dehydrogenase (GAPDH, 60004-1-Ig, Proteintech), actin (20536-1-AP, Proteintech), PGC1α (ab191838, Abcam), CPT1A (ab128568, Abcam), succinate dehydrogenase complex iron sulphur subunit B (SDHB, 10620-1-AP, Proteintech), cytochrome c oxidase subunit IV (COXIV, ab202554, Abcam), and ATPB (17247-1-AP, Proteintech). The membrane was then incubated with the corresponding secondary antibody for 1.5 h at room temperature and washed three times. The images were analysed using ImageJ software.

### Quantitative real-time polymerase chain reaction (RT-qPCR)

First, RNA was extracted from the lysed cells or kidney tissue using Trizol reagent (Invitrogen), and then the RNA was synthesized to cDNA according to the manufacturer’s instructions using the ReverTra Ace qPCR RT kit (Toyobo). Quantitative real-time polymerase chain reaction was performed using SYBR Select Master Mix (Life Technologies) and an RT-qPCR detection system (ABI). The primers for the following genes were synthesized: *PPARα* forward TTTGCCAAGGCTATCCCAGG, reverse GTCACAGAACGGCTTCCTCA,

*PGC1α* forward AATGCAGCGGTCTTAGCACT, reverse CTGAGCAGGGACGTCTTTGT, *ACOX2* forward TCATCCAACGTGACCCAGTG, reverse CAGCAAGGACTCTGTCAGCA, *CPT2* forward CATCGTACCCACCATGCACT, reverse CTCCTTCCCAATGCCGTTCT, *18S* forward AGCTATCAATCTGTCAATCCTGTC, reverse GCTTAATTTGACTCAACACGGGA, and the expression of each target gene was normalized by the *18S* gene and calculated by the 2^−ΔΔCT^ method.

### Measurement of mitochondrial membrane potential

The mitochondrial membrane potential (MMP) was detected using JC-1 (Beyotime). First, JC-1 staining working solution was obtained by adding 8 mL ultrapure water for every 50 μL of JC-1 (200X), which was vortexed violently to fully dissolve, and then 2 mL of JC-1 staining buffer (5X) was added. Second, cells in six-pore plate were washed twice in PBS, and then 1 mL of JC-1 staining working solution was added and left for 20 min at 37° C. Finally, the cells were washed with PBS again, and 2 mL of the cell culture solution was added. Visualized images were acquired using fluorescence microscopy.

### Mitochondrial reactive oxygen species (ROS) detection

Mitochondrial ROS generation was measured by fluorescence microscopy using Mito-SOX Red (Thermo Fisher Scientific). MitoSOX reagent working solution (2.0 mL, 5 μM) was added to cover the cells in the six-pore plates, followed by staining with Mito-SOX Red for 10 min at 37 °C. Then, the cells were washed with PBS three times, and the visualized images were acquired using fluorescence microscopy.

### ATP detection

ATP production was determined through the Enhanced ATP Assay Kit (Beyotime, China, Cat. No: S0027) according to the manufacturer’s protocol.

### Statistical analysis

The quantification and graphs were analysed using GraphPad Prism 7 (La Jolla, CA, USA) and presented as mean ± standard error of the mean. Differences between multiple groups were analysed using analysis of variance (ANOVA), and statistical significance was set at *P* < 0.05.

## Results

### Characterization of EVs

The isolated EVs from the culture medium of hP-MSCs were characterized as follows: both Norm-EVs and Hypo-EVs exhibited typical double-layered membrane vesicles under TEM (Fig. 1b), and their size distribution was evaluated by DLS, among which the EVs diameter ranged from ∼25 to ∼280 nm (Fig. 1c). Western blots revealed positive markers of EVs (Alix and TSG101) in the Norm-EVs and Hypo-EVs, while GM130 (a negative marker) was not detectable in these EVs (Fig. 1d).

## Internalization of EVs in vivo

**Fig. 2:**
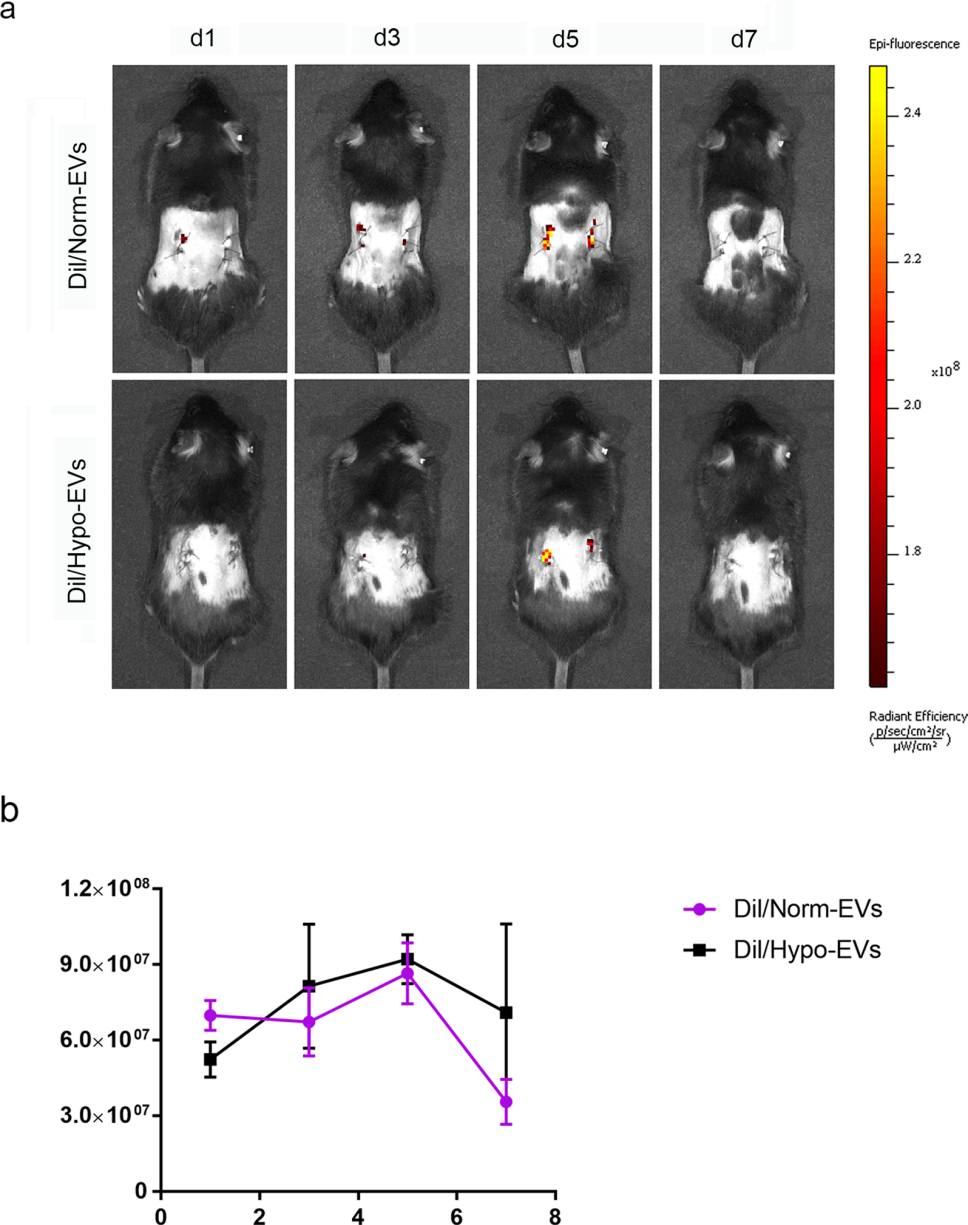
Norm-EVs and Hypo-EVs were internalized by injured kidneys. **a** Representative fluorescence images of I/R mice after treatment with Dil/Norm-EVs or Dil/Hypo-EVs at selected time points. **b** Dynamic changes in the fluorescence intensity of I/R kidneys with intravenous injection of Dil/Norm-EVs or Dil/Hypo-EVs at selected times (*n* = 3)

After characterisation of the MSC-EVs, we next tested whether EVs could be internalized into injured kidney in vivo. First, the I/R renal fibrosis models were built as mentioned above. Then, 0.15 mL of 100 μg Dil/Norm-EVs or Dil/Hypo-EVs was injected into the mice through the tail vein on the day of surgery and the next day, followed by monitoring the EVs distribution at different time points. Fluorescence signals were observed in the renal area and gradually increased from D 1, peaked on D 5 and began to decline on D 7 (Fig. 2a). Subsequent signal analysis of the kidney revealed that the fluorescence signal of the Norm-EVs was roughly the same as that of the Hypo-EVs group (Fig. 2b). Collectively, these results indicated that both Norm-EVs and Hypo-EVs could target the kidney injury sites and there were no difference in their fluorescence signal intensity.

### Hypo-EVs have a better therapeutic effect on the recovery of renal fibrosis after renal I/R than Norm-EVs

**Fig. 3:**
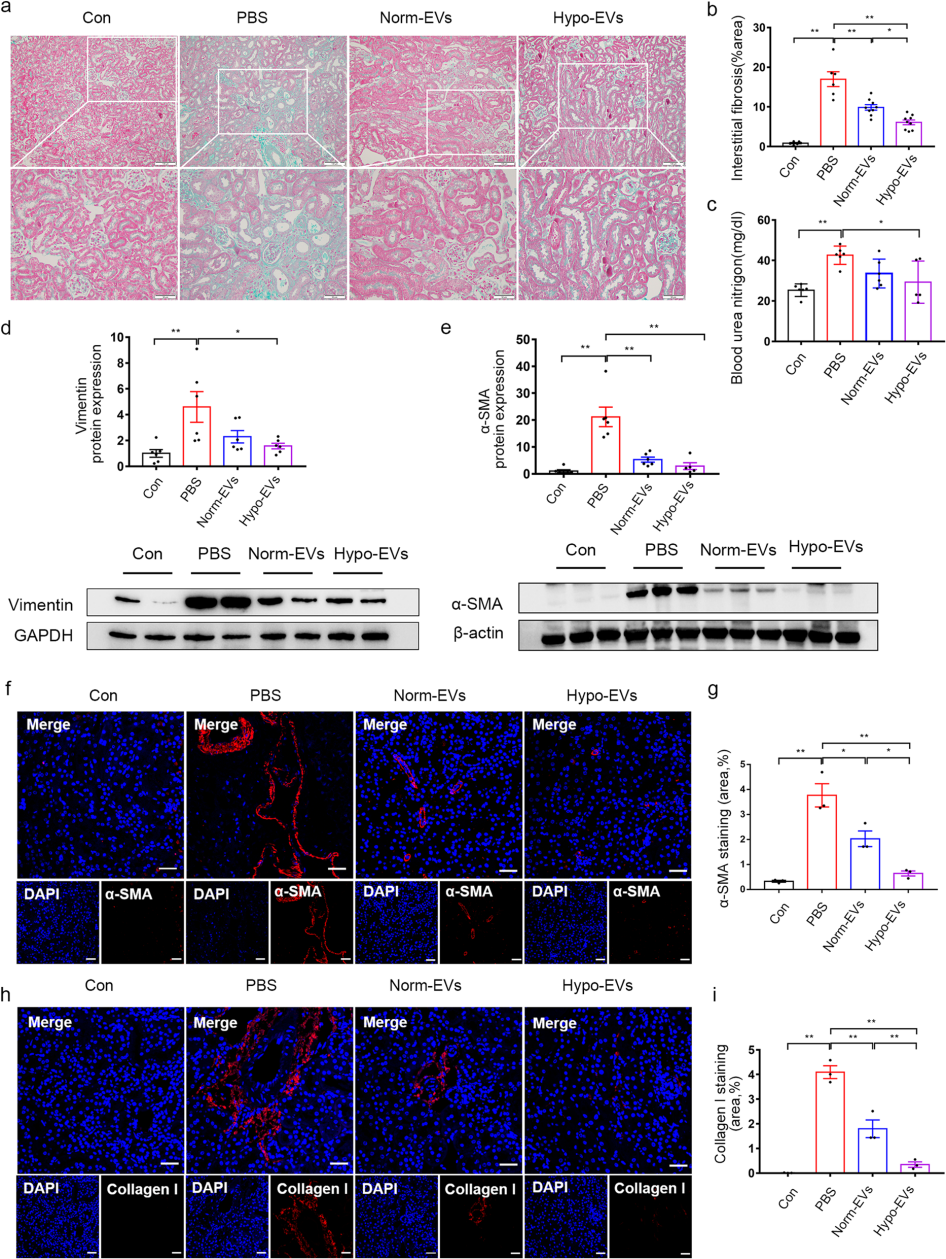
Hypo-EVs have a better anti-fibrosis effect than Norm-EVs in I/R-induced renal fibrosis mice. **a** Representative images for Masson’s staining of kidney sections on D 14 after I/R injury. **b** Quantification of interstitial fibrosis area as percentages of the total area (*n* = 6–9). **c** Renal function analysis. Levels of BUN in mice on D 3 after I/R injury (*n* = 5–6). **d** Western blot analysis was used to detect the protein expression of vimentin. The relative expression of vimentin was evaluated (*n* = 5–6). **e** Western blot analysis was used to detect the protein expression of α-SMA. The relative expression of α-SMA was evaluated (*n* = 5–6). **f** Immunofluorescence staining of α-SMA (red) and nuclei (blue). **g** Quantification of α-SMA-positive areas as percentages of the total area (*n* = 3). **h** Immunofluorescence staining of collagen I (red) and nuclei (blue). **i** Quantification of collagen I-positive areas as percentages of the total area (*n* = 3). Scale bar represents 50 μm. Data are expressed as mean ± SEM. *P < 0.05 and **P < 0.01

After confirming that MSC-EVs could target the injured kidney, we used Masson’s staining to evaluate the pathological changes at D 2, D 7 and D 14 after renal I/R injury. Masson trichrome staining showed only slight interstitial fibrosis occurs at D 2 and D 7 after I/R injury. Further progression of interstitial fibrosis and tubular atrophy were observed at D 14 after I/R injury (Additional file 1). We observed fewer tubulointerstitial injuries and tubular atrophy in the Norm-EVs and Hypo-EVs groups than in the other groups, and further suppression was observed in the Hypo-EVs group at D 14 (Fig. 3a, b). So we considered that the mice model at D 14 after I/R injury was suitable for evaluating the anti-fibrotic effect of MSC-EVs. To further validate the therapeutic efficacy of our treatment, the levels of blood urea nitrogen, an indicator of renal function, was detected by Urea Assay Kit. Notably, Hypo-EVs, but not Norm-EVs, further suppressed the level of BUN in the serum compared with the PBS group, which indicated that Hypo-EVs could improve renal function decline induced by I/R (Fig. 3c). Similarly, I/R injury significantly increased the expression of vimentin and only administration of Hypo-EVs suppressed the protein expression levels of vimentin compared with the PBS group (Fig. 3d). Immunohistochemistry also revealed that the vimentin-positive area was reduced in the Hypo-EVs group compared with the PBS group (Additional file 2). The protein expression levels of α-SMA were also markedly decreased in the Norm-EV and Hypo-EV groups (Fig. 3e). In addition, we detected collagen I and α-SMA expression by immunofluorescence. The expression of collagen I and α-SMA were significantly higher than in the PBS group. Moreover, collagen I and α-SMA expression in the kidney were significantly decreased after Norm-EVs and Hypo-EVs treatment. A more obvious reduction in collagen I and α-SMA were observed by administration of Hypo-EVs (Fig. 3f–i). Overall, these results indicate that Hypo-EVs have a better anti-fibrosis effect than Norm-EVs in I/R-induced kidney damage.

### Hypo-EVs prevents impaired CPT1A-mediated FAO in I/R-induced renal fibrosis model

**Fig. 4:**
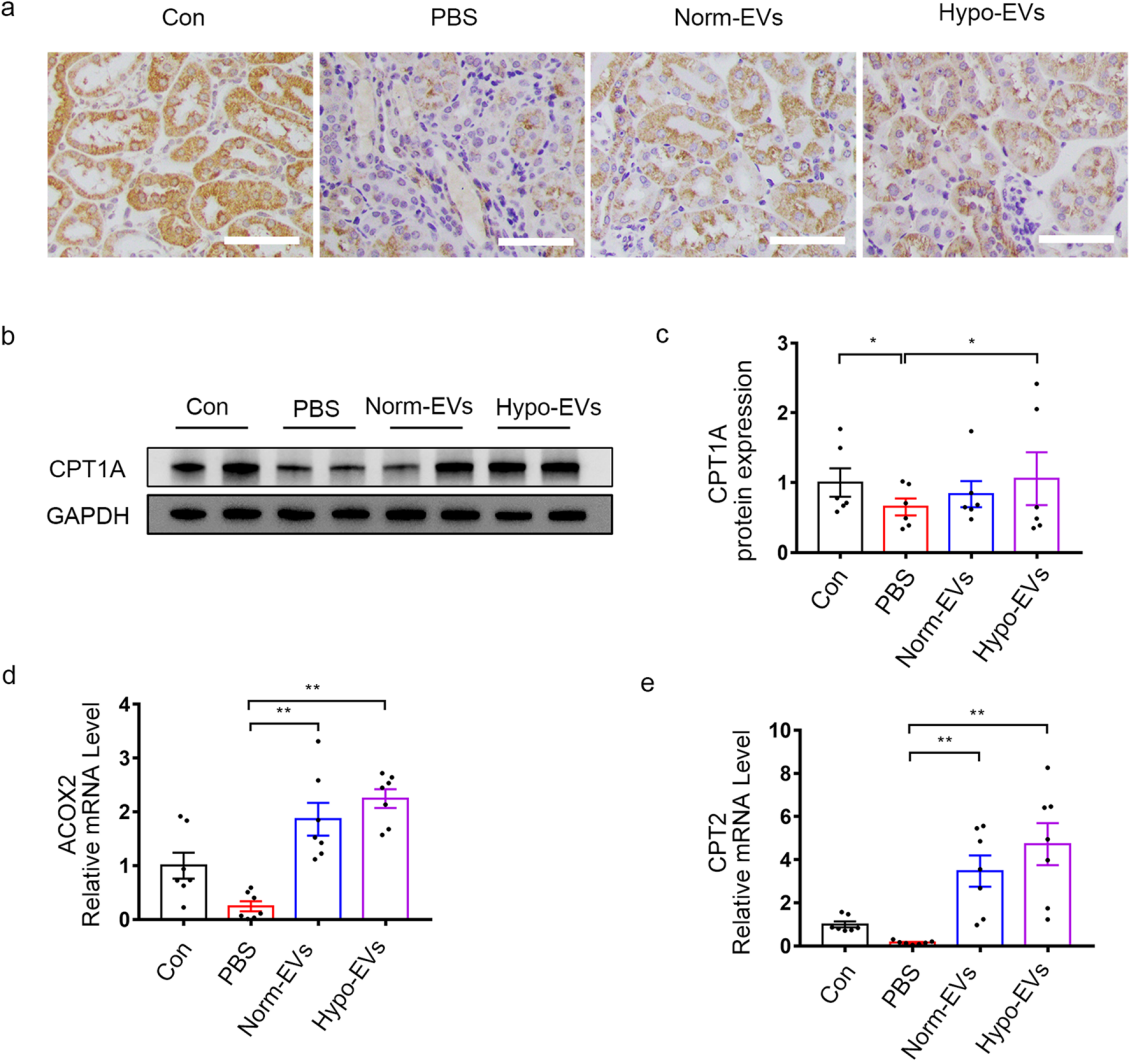
Hypo-EVs reverse CPT1A-mediated FAO defects in I/R-induced fibrotic kidneys. **a** The locations and expressions of CPT1A were determined by immunohistochemical staining in the kidney sections of mice from the different groups. **b**, **c** Western blot analysis was used to detect the protein expression of CPT1A. The relative expression of CPT1A was evaluated (*n* = 6). **d** The mRNA level of ACOX2 in different groups (*n* = 6–7). **e** The mRNA level of CPT2 in different groups (*n* = 7). Scale bar represents 50 μm. Data are expressed as mean ± SEM. *P < 0.05 and ** *P* < 0.01

It has been reported that FAO deficiency is closely related to the progression of renal fibrosis, and restoring impaired FAO could be an effective strategy to alleviate kidney fibrosis [20]. CPT1 is the key rate-limiting enzyme in FAO. Thus, we used immunohistochemical staining to test the expression of CPT1A in fibrotic kidneys, and our results showed that the expression of CPT1A in both the Norm-EVs and Hypo-EVs groups was increased. A more obvious increase in CPT1A was observed by administration of Hypo-EVs (Fig. 4a). Consistent with immunohistochemical staining, the results of immunoblot analysis showed that the protein expression levels of CPT1A were well restored after treatment with Hypo-EVs (Fig. 4b, c). Moreover, we measured the gene expression levels of other metabolic enzymes related to FAO carnitine palmitoyl transferase 2 (CPT2) and acyl-coenzyme A oxidase 2 (ACOX2), which were significantly increased after Norm-EVs and Hypo-EVs treatment, and there was no obvious difference between them (Fig. 4d,e). Taken together, these data suggest that dysregulated CPT1A-mediated FAO during kidney fibrosis can be reversed by Hypo-EVs.

### Hypo-EVs can restore PPARα and PGC1α expression, but which not involved in Hypo-EVs induced better recovery of the FAO

**Fig. 5:**
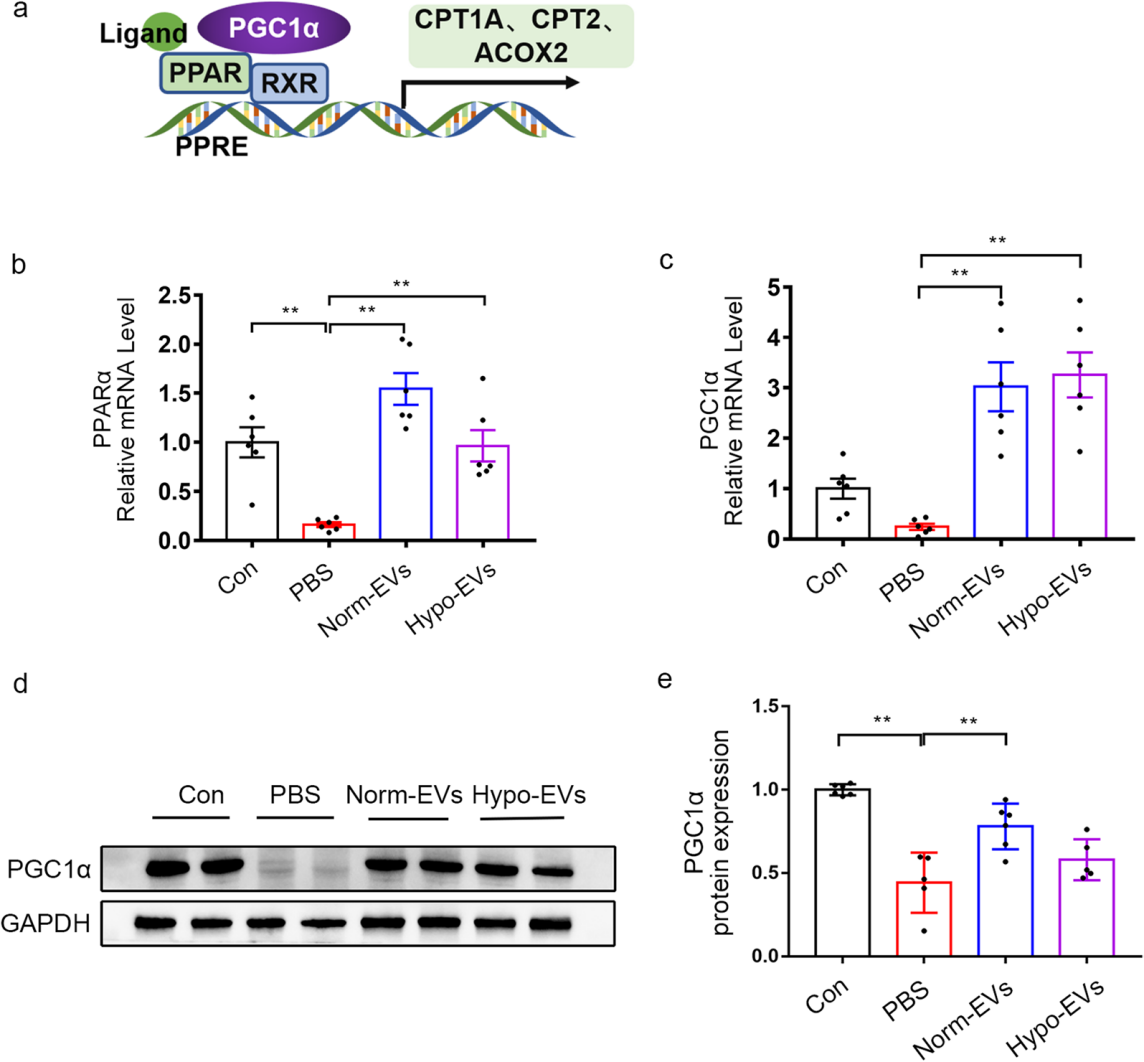
Restoration of PPARα and PGC1α expression not involved in Hypo-EVs induced better recovery of FAO. **a** Schematic illustration for FAO regulation. PPARα and PGC1α are the major regulators of FAO. Promoting the activity of PPARα can boost FAO, and PGC1α is a coactivator of PPARα. **b** The mRNA level of PPARα in different groups (*n* = 6). **c** The mRNA level of PGC1α in different groups (*n* = 6). **d**, **e** Western blot analysis was used to detect the protein expression of PGC1α. The relative expression of PGC1α was evaluated. Data are expressed as mean ± SEM. *P < 0.05 and ** *P* < 0.01

Peroxisome proliferators-activated receptors (PPARs) are members of the nuclear receptor superfamily of transcription factors that regulate metabolic processes of fatty acid metabolism and are activated by endogenous or exogenous ligands, such as fatty acids and their derivatives or synthetic agonists. Activated PPAR forms a heterodimer with the coactivator retinoid x receptor (RXR) and subsequently assembles at specific DNA response elements called PPAR response elements, resulting in transactivation of several genes related lipid metabolism [35–37]. Regulation of PPARα activity factors, including PPARα gene expression and protein translation, ligand specificity and availability, cofactor recruitment, corepressors or coactivators, and posttranslational modification, is an effective strategy to prevent and treat lipid disorders in kidney disease [38]. In particular, peroxisome proliferator-activated receptor gamma (PPAR-γ) coactivator 1-alpha (PGC1α) regulates FAO by increasing the activity of PPARα in the proximal tubule epithelial cells [39] (Fig. 5a). It has been proved that increasing the expression of PGC-1α in TECs can restore energy deficiency and prevent acute and chronic renal damage [40]. Therefore, we test the gene expression of transcription regulators related to FAO (PGC1α and PPARα). Our results indicated that the gene expression of PGC1α and PPARα were significantly increased after Norm-EVs and Hypo-EVs treatment, and there was no obvious difference between them (Fig. 5b, c). Moreover, we applied immunoblot to assess the protein expression levels of PGC1α. Our results indicated that Norm-EVs, but not Hypo-EVs, further upregulated the expression of PGC1α in the I/R-injured kidney as compared to PBS group (Fig. 5d, e). All these data suggested that Hypo-EVs did not promote recovery of the CPT1A-mediated FAO after I/R injure by restoring its major regulators PGC1α and PPARα, indicating that Hypo-EVs may work through other mechanisms to produce better recovery of the FAO.

### Hypo-EVs attenuated mitochondrial damage in renal fibrosis

**Fig. 6:**
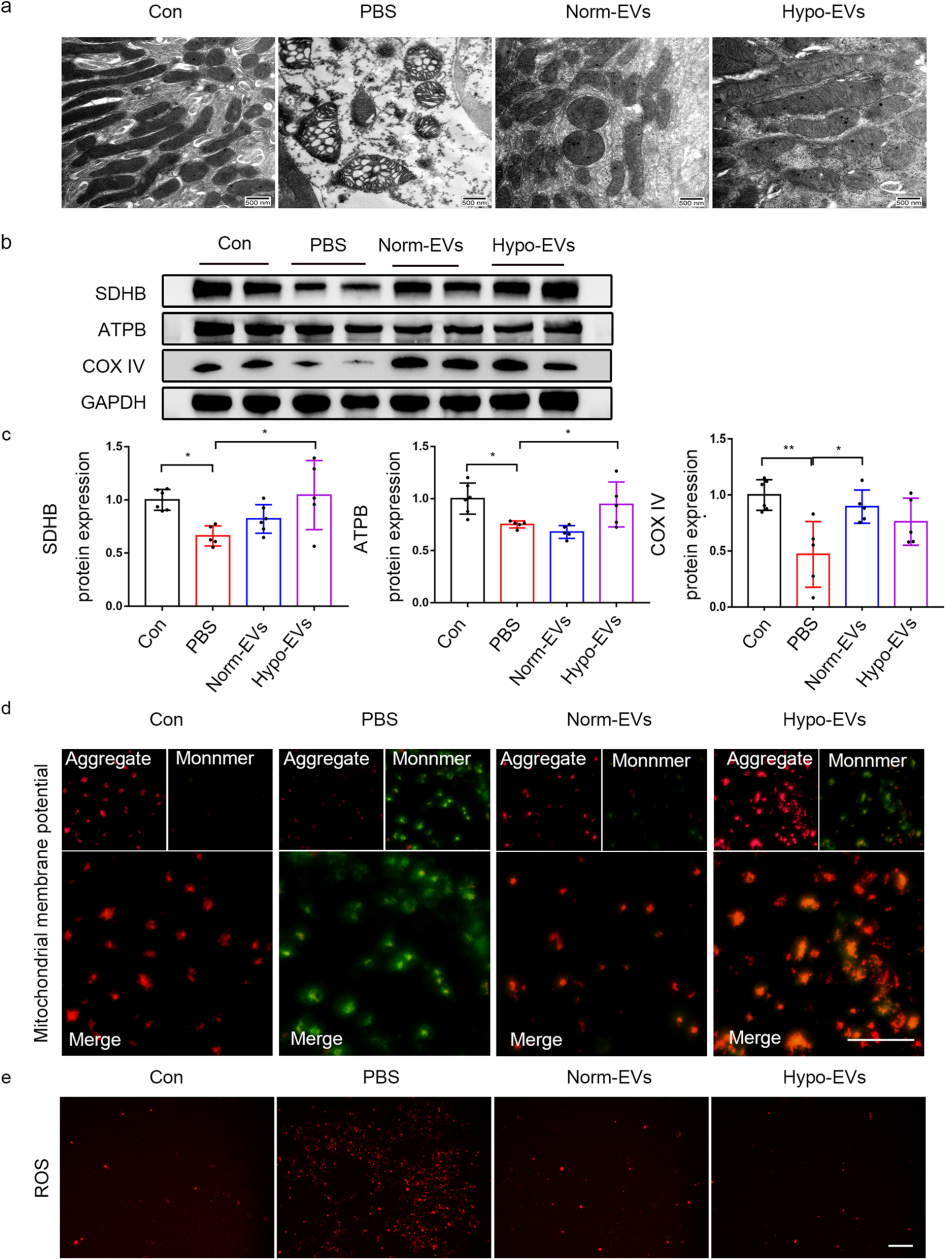
Hypo-EVs rescued mitochondrial homeostasis in vivo. **a** Representative TEM images of kidneys on D 14 after I/R injury. Scale bar represents 500 nm. **b**, **c** Western blot was used to detect the protein expression of mitochondrial OXPHOS proteins (ATPB、SDHB and COX IV). The relative expression of ATPB, SDHB and COX IV were evaluated (*n* = 5–6). **d** Mitochondrial membrane potential was determined through a JC-1 probe in HK-2 cells after incubated with TGF-β1. Scale bar represents 100 μm. **e** Mito-SOX Red was used to observe the levels of intracellular ROS in the HK-2 cells after incubated with TGF-β1. Scale bar represents 100 μm. Data are expressed as mean ± SEM. *P < 0.05,** < 0.01

Considering that mitochondria play an indispensable role in regulating FAO [41]. It has been shown that prevention of mitochondrial dysfunction during a folic acid (FA)-induced AKI event can be a useful strategy to prevent the transition to CKD [42]. In order to observe whether Hypo-EVs affect FAO by improving mitochondrial function. We used TEM to evaluate the morphological alterations of mitochondria in fibrotic kidneys. Our results showed that at D 14 after I/R injury, the number of mitochondria was decreased, and the mitochondrial structure showed severe damage, such as fragmented appearance and abnormal cristaea. As expected, the MSC-EVs treatment reversed the mitochondrial damage, and Hypo-EVs treatment better restored morphological alterations than Norm-EVs treatment (Fig. 6a). Next, we analysed mitochondrial mass by assessing the levels of mitochondrial oxidative phosphorylation (OXPHOS) proteins (ATPB、SDHB and COX IV). We found a significant decrease in mitochondrial mass after I/R injury, and Hypo-EVs treatment had a greater effect on rescuing the decreased ATPB and SDHB protein levels. Norm-EVs treatment had a better effect on the upregulation of COXIV protein expression as compared to the other treatment groups (Fig. 6b, c). In addition, the levels of ATP were decreased in fibrotic kidney tissue compared to normal kidney tissue, which was reversed after Hypo-EVs treatment (Additional file 3). To study the effect of Hypo-EVs on mitochondrial function in I/R-induced fibrosis, we measured the mitochondrial membrane potential and reactive oxygen species (ROS) changes in HK2 cells. Our results showed that mitochondrial membrane potential was repressed in HK-2 cells after incubated with TGF-β1 for 48 h and was significantly increased after Hypo-EVs treatment (Fig. 6d). The same results were observed in the cell hypoxia/reoxygenation (H/R) model (Additional file 4). Besides that, Hypo-EVs treatment downregulated levels of intracellular ROS in HK2 cells incubated with TGF-β1 (Fig. 6e). These results demonstrated that Hypo-EVs treatment could promote recovery of the CPT1A-mediated FAO partly by recovering mitochondrial homeostasis.

## Discussion

**Fig. 7:**
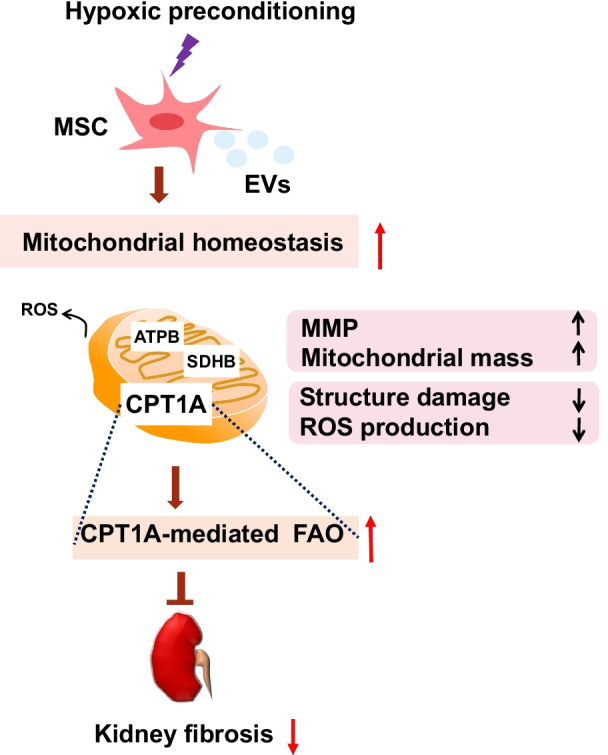
Possible mechanisms for preventing renal fibrosis using hypoxic mesenchymal stem cell-derived extracellular vesicles (Hypo-EVs)

In this study, we focused on the FAO in renal fibrosis, and we identified metabolic enzymes(CPT1A) and transcription regulators (PGC1α, PPARα) of FAO, which indicated that Hypo-EVs can reverse fatty acid metabolism disorder in fibrotic kidney caused by I/R. Moreover, we found that Hypo-EVs exert a profound effect on CPT1A-mediated FAO, which partly attributed to the regulation of mitochondrial homeostasis (Fig. 7).

In recent years, an increasing number of studies have reported the renal protective effect of MSC-EVs in vivo models of CKD, and a small amount of available data demonstrated that EVs were superior to or equally effective in renoprotection compared with their parent MSC [43]. The treatment of MSC-EVs in CKD is in its infancy, and the complex mechanism has not been fully demonstrated. AKI is one of the major factors of CKD, which leads to end-stage renal disease [44], and renal I/R injury is a major factor for AKI, so we select I/R induced fibrosis model to uncover potential molecular mechanism that how MSC-EVs prevents against renal fibrosis.

The low yield of MSC-EVs is a limiting factor for large-scale production of cell-free therapies. Thus, various potential approaches to increase the yield of EVs have been studied, including optimisation of MSC culture conditions, three-dimensional extracellular matrix-based scaffolds, and regulation of EVs biogenesis [10]. Among them, hypoxic conditioning of MSC is a valid method to augment the effect of EVs treatment in various diseases, such as bone fracture healing [30], diabetic wound healing [29], and myocardial repair [45]. Our study provides evidence for hypoxia-enhanced action of EVs secreted by MSC. Moreover, we also show that hypoxic preconditioned MSC-derived EVs have a greater anti-fibrosis and renal protective effect than Norm-EVs in CKD induced by I/R.

Data on miRNAs in Hypo-EVs compared to Norm-EVs have been well reported. Numerous studies have reported that miRNA content was responsible for the beneficial effects of Hypo-EVs in many diseases [30, 46]. Compared with Norm-EVs, 215 miRNAs were upregulated and 369 miRNAs downregulated in adipose derived Hypo-EVs, which mainly regulate cell metabolism, differentiation and TGF-β function [29]. However, the mechanism by which Hypo-EVs regulate metabolism function remains poorly understood in CKD. A large body of the literature indicates that FAO is reduced in kidney fibrosis and contributes to its pathogenesis [47]. Consistent with the reports, our study showed that the gene expression of FAO-related key metabolic enzymes (CPT1A, CPT2, and ACOX2) and the FAO transcription regulatory factors (PPARα and PGC1α) were markedly decreased in renal fibrosis induced by I/R. Moreover, restoration of lipid metabolism is a potential strategy for treating renal fibrosis [48]. The overexpression of CPT1A in kidney tubules is a significant contributor to gain of function in FAO and results in protection of renal function and fibrosis by preventing mitochondrial dysfunction, TEC differentiation, and inflammation in CKD animal models [21]. Our data showed that Hypo-EVs could well reverse damaged FAO in I/R-induced fibrosis, which suggests that the therapeutic effects of Hypo-EVs in renal fibrosis are partly the result of restoring FAO.

Impairment renal PPARα signalling decreased activity of the FAO pathway and aggravated renal fibrosis development [49]. Administration of fenofibrate, a PPARα agonist, restored the fatty oxidation defect and tubulointerstitial fibrosis in CKD [50, 51]. PGC-1α is a coactivator of PPARα in the transcriptional control of mitochondrial FAO capacity [39]. We investigated whether Hypo-EVs can increase PPARα signaling and restore FAO in I/R injury. Our results showed that there were no obvious difference in the expression of PGC-1α and PPARa between Norm-EVs and Hypo-EVs. Therefore, there must be other factors that promote the occurrence of this process. Mitochondrias are the powerhouse for FAO to generate energy for the cell [52]. Mitochondrial dysfunction is critical in the pathogenesis of kidney fibrosis [53]. Restoration of mitochondrial homeostasis has emerged as a promising therapeutic strategy to prevent kidney injury and accelerate kidney repair [25]. Recent studies have indicated that MSC-EVs have a protective effect on mitochondrial damage caused by AKI, which protects TECs against insult by reducing mitochondrial fragmentation, normalising mitochondrial membrane potential, and reversing mtDNA deletion and mitochondrial OXPHOS defects [26, 27]. Our results showed that Hypo-EVs treatment recovered abnormal mitochondrial morphology shift, increased mitochondrial mass and enhanced mitochondrial function in fibrotic kidneys. Our findings indicate that effects of Hypo-EVs on FAO may be achieved by regulating mitochondrial homeostasis.

There are some limitations in our study. For example, our data demonstrate that Hypo-EVs have better anti-fibrosis effects in I/R injury mice than Norm-EVs. However, we did not determine the differentially expression of contents between Norm-EVs and Hypo-EVs and also not evaluated the specific manner by which Hypo-EVs affect mitochondrial homeostasis in this study.

## Conclusions

In summary, our data indicate that Hypo-EVs significantly inhibit the renal fibrosis by restoring CPT1A-mediated FAO, and these effects may be achieved by regulating mitochondrial homeostasis. Our findings provide further mechanism support for the development of cell-free therapy for renal fibrosis.

## Supplementary Information

**Additional file 1: **Renal interstitial fibrosis development after I/R injure.

**Additional file 2: **Representative images of immunohistochemical staining for vimentin.

**Additional file 3: **The ATP levels in different groups.

**Additional file 4: **Hypo-EVs rescued mitochondrial membrane potential in HK2 cells after hypoxia/reoxygenation injury.

## Data Availability

The datasets and resources generated and analysed during the current study are available from the corresponding author upon reasonable request.

## Abbreviations

MSC-EVs: Mesenchymal stem cell-derived extracellular vesicles
CKD: Chronic kidney disease
PTC: Proximal tubular cell
FAO: Fatty acid oxidation
I/R: Ischemia–reperfusion
CPT1A: Carnitine palmitoyl-transferase 1A
PGC1α: Peroxisome proliferator-activated receptor gamma coactivator 1-alpha
PPARα: Peroxisome proliferators-activated receptors alpha
TECs: Thymic epithelial cells
ATP: Adenosine triphosphate
AKI: Acute kidney injury
hP-MSCs: Human placenta-derived MSCs
DMEM/F12: Dulbecco’s modified eagle’s medium/nutrient mixture F-12
HK-2: Human renal proximal tubular epithelial
TGF-β1: Transforming growth factor beta-1
BCA: Bicinchoninic acid protein assay
TEM: Transmission electron microscopy
DLS: Dynamic light scattering
BLI: Bioluminescence imaging
ROIs: Regions of interest
BUN: Blood urea nitrogen
DAB: 3,3’-Diaminobenzidine
RIPA: Radio-immune precipitation assay
PMSF: Phenylmethylsulphonyl fluoride
SDS-PAGE: Sodium dodecyl sulphate–polyacrylamide gel
α-SMA: α-Smooth muscle actin
GADPH: Glyceraldehyde 3-phosphate dehydrogenase
SDHB: Succinate dehydrogenase complex iron sulphur subunit B
COXVI: Cytochrome c oxidase subunit IV
RT-qPCR: Quantitative real-time polymerase chain reaction
ROS: Reactive oxygen species
PBS: Phosphate-buffered saline
ANOVA: Analysis of variance
CPT2: Carnitine palmitoyl transferase 2
ACOX2: Acyl-coenzyme A oxidase 2
OXPHOS: Oxidative phosphorylation
IHC: Immunohistochemistry
