# Supplementary material for: Hypoxic mesenchymal stem cell-derived extracellular vesicles ameliorate renal fibrosis after ischemia–reperfusion injure by restoring CPT1A mediated fatty acid oxidation
Source: Stem Cell Res Ther. 2022 May 7;13:191. doi: 10.1186/s13287-022-02861-9 (PMC9080148; doi:10.1186/s13287-022-02861-9)
Supplement: Supplementary file 1 — Additional file 1: Renal interstitial fibrosis development after I/R injure. [file 13287_2022_2861_MOESM1_ESM.pdf]

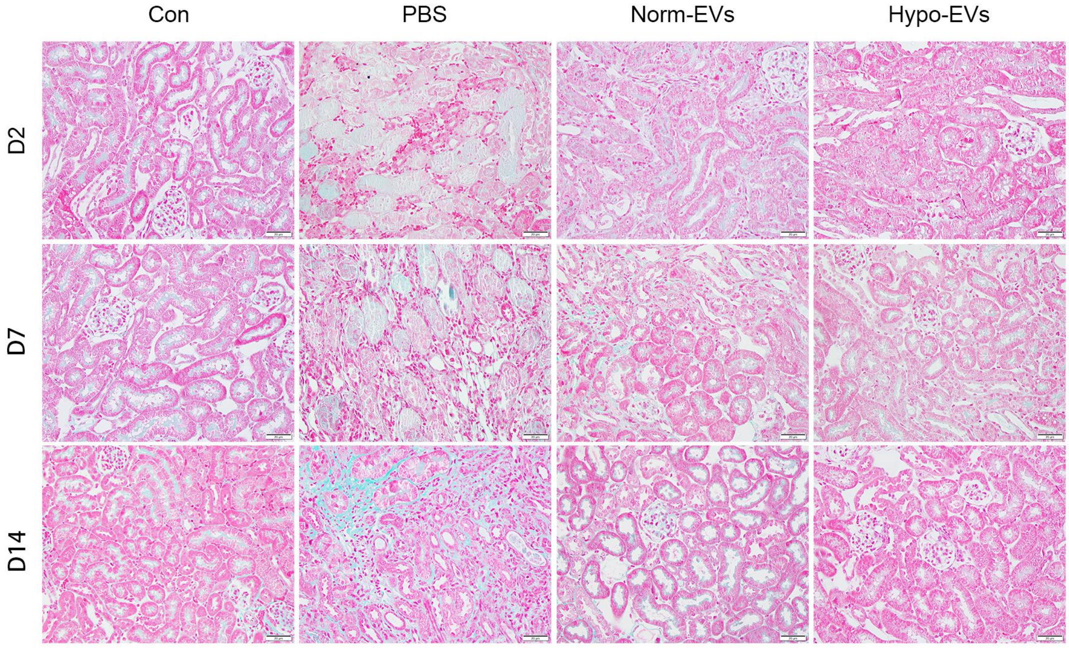

Additional file 1 Renal interstitial fibrosis development after I/R injure

Representative images of Masson trichrome staining in kidney at D2, D7 and D14 after I/R injure. Scale bar represents 50 μm.
