# Supplementary material for: Hypoxic mesenchymal stem cell-derived extracellular vesicles ameliorate renal fibrosis after ischemia–reperfusion injure by restoring CPT1A mediated fatty acid oxidation
Source: Stem Cell Res Ther. 2022 May 7;13:191. doi: 10.1186/s13287-022-02861-9 (PMC9080148; doi:10.1186/s13287-022-02861-9)
Supplement: Supplementary file 2 — Additional file 2: Representative images of immunohistochemical staining for vimentin. [file 13287_2022_2861_MOESM2_ESM.pdf]

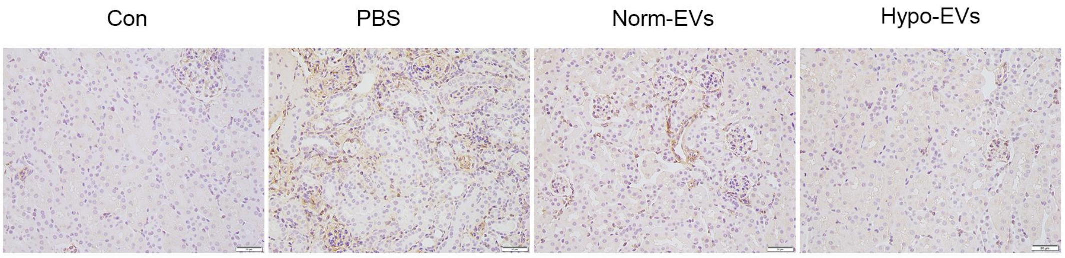

Additional file 2 Representative images of immunohistochemical staining for vimentin.

The locations and expressions of vimentin was determined by immunohistochemical staining in the kidney sections of mice from the different groups. Scale bar represents 50μm.
