# Supplementary material for: Hypoxic mesenchymal stem cell-derived extracellular vesicles ameliorate renal fibrosis after ischemia–reperfusion injure by restoring CPT1A mediated fatty acid oxidation
Source: Stem Cell Res Ther. 2022 May 7;13:191. doi: 10.1186/s13287-022-02861-9 (PMC9080148; doi:10.1186/s13287-022-02861-9)
Supplement: Supplementary file 3 — Additional file 3: The ATP levels in different groups. [file 13287_2022_2861_MOESM3_ESM.pdf]

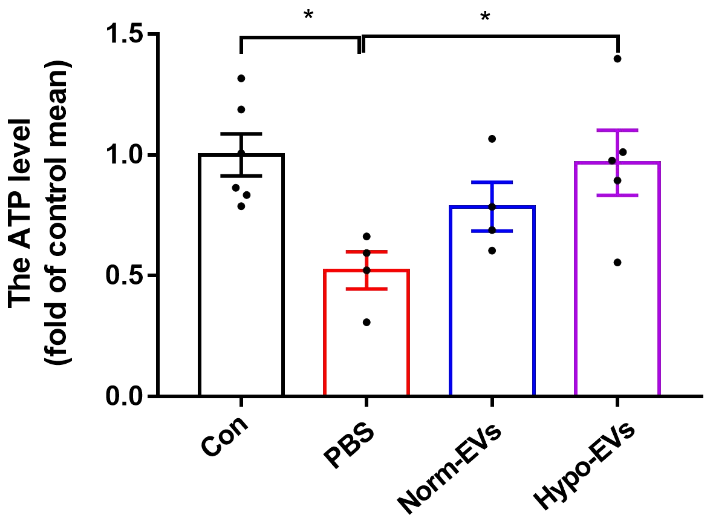

Additional file 3 The ATP levels in different groups

ATP production was measured through Enhanced ATP Assay Kit. Data are expressed as mean ± SEM. *P < 0.05 and **P < 0.01.
