# Supplementary material for: Hypoxic mesenchymal stem cell-derived extracellular vesicles ameliorate renal fibrosis after ischemia–reperfusion injure by restoring CPT1A mediated fatty acid oxidation
Source: Stem Cell Res Ther. 2022 May 7;13:191. doi: 10.1186/s13287-022-02861-9 (PMC9080148; doi:10.1186/s13287-022-02861-9)
Supplement: Supplementary file 4 — Additional file 4: Hypo-EVs rescued mitochondrial membrane potential in HK2 cells after hypoxia/reoxygenation injury. [file 13287_2022_2861_MOESM4_ESM.pdf]

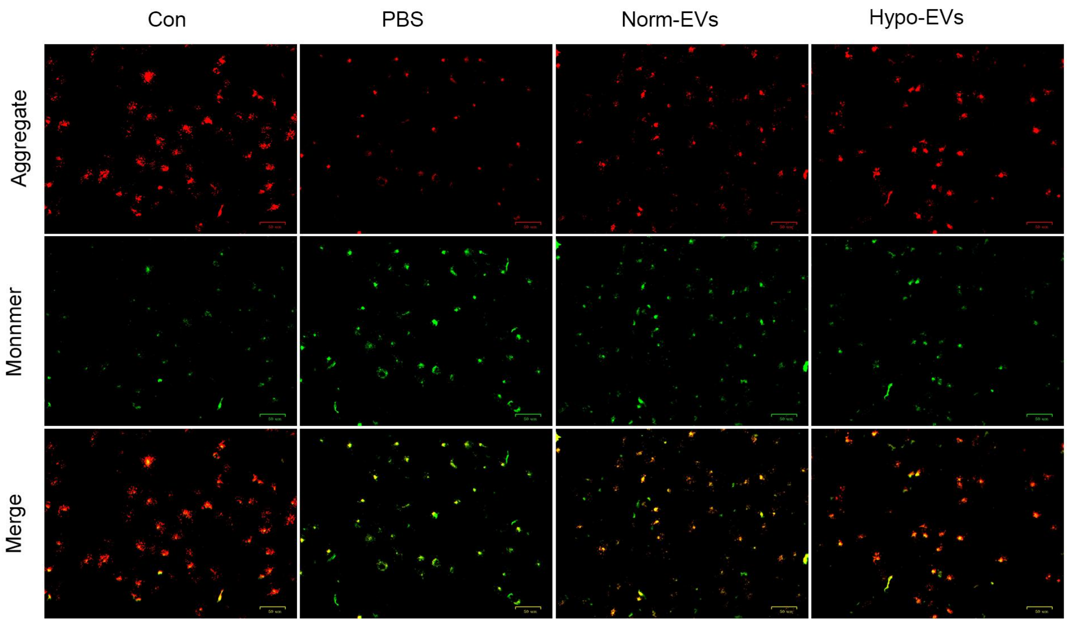

Additional file 4 Hypo-EVs rescued mitochondrial membrane potential in HK2 cells after hypoxia/reoxygenation injury.

The mitochondrial membrane potential was determined through a JC-1 probe in HK-2 cells. Scale bar represents 50 μm.
